# How Tyramine β-Hydroxylase Controls the Production of Octopamine, Modulating the Mobility of Beetles

**DOI:** 10.3390/ijms19030846

**Published:** 2018-03-14

**Authors:** Li Xu, Hong-Bo Jiang, Xiao-Feng Chen, Ying Xiong, Xue-Ping Lu, Yu-Xia Pei, Guy Smagghe, Jin-Jun Wang

**Affiliations:** 1 Key Laboratory of Entomology and Pest Control Engineering, College of Plant Protection, Southwest University, Chongqing 400715, China; Xuli940208@163.com (L.X.); jhb8342@swu.edu.cn (H.-B.J.); 18883375009@163.com (X.-F.C.); xiongying842620@163.com (Y.X.); luxueping91@163.com (X.-P.L.); pyx20830@163.com (Y.-X.P.); guy.smagghe@ugent.be (G.S.); 2 Academy of Agricultural Sciences, Southwest University, Chongqing 400715, China; 3 Department of Crop Protection, Ghent University, 9000 Ghent, Belgium

**Keywords:** *Tribolium castaneum*, octopamine, tyramine-β-hydroxylase, mobility

## Abstract

Biogenic amines perform many kinds of important physiological functions in the central nervous system (CNS) of insects, acting as neuromodulators, neurotransmitters, and neurohormones. The five most abundant types of biogenic amines in invertebrates are dopamine, histamine, serotonin, tyramine, and octopamine (OA). However, in beetles, an important group of model and pest insects, the role of tyramine β-hydroxylase (TβH) in the OA biosynthesis pathway and the regulation of behavior remains unknown so far. We therefore investigated the molecular characterization and spatiotemporal expression profiles of TβH in red flour beetles (*Triboliun castaneum*). Most importantly, we detected the production of OA and measured the crawling speed of beetles after ds*TcTβH* injection. We concluded that *TcTβH* controls the biosynthesis amount of OA in the CNS, and this in turn modulates the mobility of the beetles. Our new results provided basic information about the key genes in the OA biosynthesis pathway of the beetles, and expanded our knowledge on the physiological functions of OA in insects.

## 1. Introduction

Biogenic amines perform many kinds of important physiological functions in the central nervous system (CNS) of insects, acting as neuromodulators, neurotransmitters, and neurohormones [1]. The five most abundant types of biogenic amines in invertebrates are dopamine (DA), histamine (HA), serotonin (5-HT), tyramine (TA), and octopamine (OA) [2]. The characterization and function of individual aminergic systems have been studied in many insect species. It has been reported that OA could influence avoidance learning [3] and modulate the behavior of stinging [4] and feeding [5] in the honeybee. In crickets, the octopaminergic system could influence the physical exertion of fighting and some rewarding aspects of the winning experience, leading to a transient increase in aggressive motivation [6]. In locusts, OA plays an important role in flight activity [7] and olfactory attraction [8]. In *Tribolium freemani,* OA was reported to modulate the activity of the juvenile hormone esterase [9]. It has been previously suggested that OA could modulate various behaviors in the model insect *Drosophila melanogaster* [1]. OA is necessary and sufficient for exercise adaptation in *Drosophila* [10] and plays an important role in regulating fitness [11]. In addition, OA is required for modulating crawling in the larvae of *Drosophila* [12].

The biosynthesis process of OA in insects has been well documented. This process consists of two steps: decarboxylation of tyrosine to tyramine [13], and hydroxylation of tyramine to OA [14]. During this process, the tyramine β-hydroxylase (TβH) is a key enzyme involved in the hydroxylation of tyramine, which seems to be extraordinarily important in controlling the production of OA in the nervous system.

Recently, several research groups have looked at the relationship between insect TβH and OA production [15]. The increased activity of TβH in the brain with elevated OA production during different developmental stages of *Manduca sexta* has been documented [16]. In honey bees, the expression of TβH increased to produce more OA in the brain when they were foraging [17]. In *Drosophila*, TβH-mutant individuals produced fewer rhythmic bursts and waves of body-wall contractions [18].

Although the function of the octopaminergic system has been extensively studied in many insects, its physiological function is barely known in beetles. In order to investigate the effect of OA production on beetles’ behavior, we used the red-flour beetle (*Tribolium castaneum*), an important model and pest insect, targeting the key enzyme involved in the OA biosynthesis pathway. qRT-PCR was applied to investigate its developmental and spatial expression patterns and the double-stranded RNA (dsRNA) injection-mediated RNA interference (RNAi) was used to knockdown *TcTβH*. The HPLC was further employed to detect OA production after RNAi of *TcTβH*. The crawling speed of adult *T. castaneum* was also measured after RNAi. We found that TβH regulated OA production, which modulates the mobility of *T. castaneum*. Our results will not only add basic information on the physiological function of OA in the beetles, but also help to elucidate the complex network regulating the locomotion of insects.

## 2. Results

### 2.1. Molecular Cloning and Sequences Analysis

**Figure 1:**
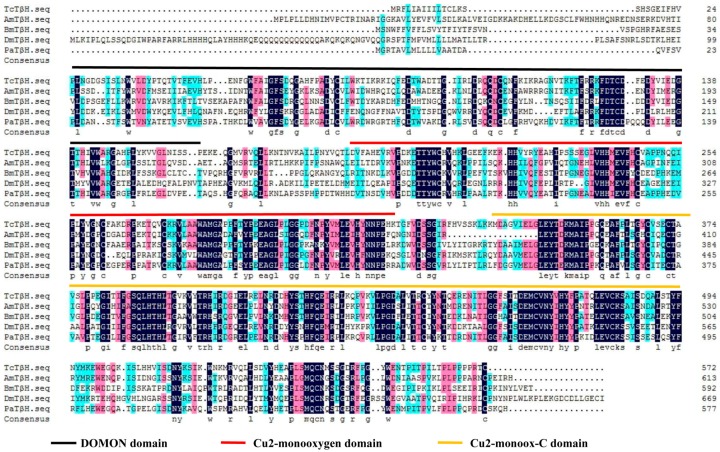
Multiple sequence alignment of TcTβH from four insect species: *Apis mellifera* (AmTβH), *Drosophila melanogaster* (DmTβH), *Bombyx mori* (BmTβH) and *Periplaneta americana* (PaTβH), by DNAMAN. The three different color lines represent the conserved domains, the black line is the DOMON domain, the red line the Cu2-monooxygen domain and the yellow line the Cu2-monoox-C domain. The information on TβH in these species is listed in Table S2.

**Figure 2:**
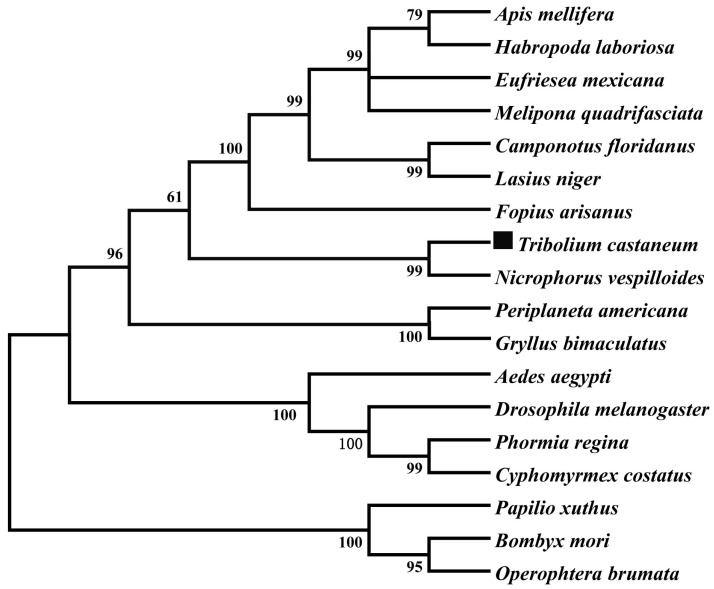
The phylogenetic tree of TcTβH (marked by black square) and various amino acids of the TβH neighbor-joining tree were constructed in MEGA 5 using 1000 bootstrap tests re-sampling. The numbers at the nodes of the branches represent the level of bootstrap support for each branch. The information on TβH in these species is listed in Table S2.

The TβH transcript was identified from the genome database of T. castaneum (BAM21526.1) by a tBLASTn search using the TβH transcript of D. melanogaster (NP-788884.1). The full-length cDNA sequence of TcTβH is 1799 bp with an open reading frame (ORF) of 1721 bp, which encodes a protein of 572 amino acids. The BLAST search with National Center for Biotechnology Information (NCBI) (www.ncbi.nlm.nih.gov/Structure/cdd/wrpsb.cgi) showed that TcTβH has three different conserved domains (Figure 1), including a DOMON domain (from 24 to 162), a copper type II ascorbate-dependent monooxygenase (Cu2-monooxygenase) located at the N-terminal domain from position 190 to 318, and a copper type II ascorbate-dependent monooxygenase (Cu2-monoox-C) located at the C-terminal domain from position 338–494. Multiple sequence alignments with TβH from several other insect species showed that the domains of TβH were highly conserved (Figure 2). Furthermore, the phylogenetic tree constructed with TβHs in 18 insect species showed that TβHs were grouped based on the relationships among species. According to the tree, TcTβH clustered with NvTβH (from Nicrophorus vespilloides, AHN85840.1) and a BLASTP search of the NCBI databases found that the similarity was 67%.

### 2.2. Expression Profiles

**Figure 3:**
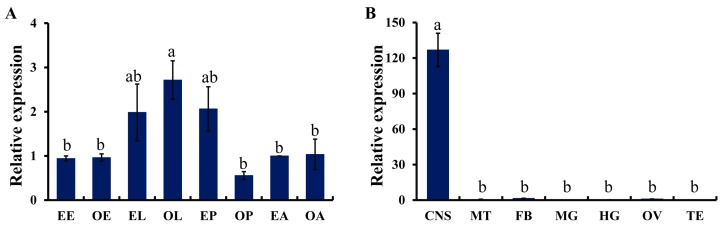
The expression profiling of *TcTβH*. (**A**) Relative expression levels of *TcTβH* at different developmental stages. The first letters, E and O represent early and old. Different stages are displayed by capitalized letters: E (egg), L (larva), P (pupa), and A (adult). The expression of EA served as the calibrator; (**B**) Relative expression levels of *TcTβH* in various tissues of adults. CNS: central nervous system; FB: fat body; MG: midgut; MT: Malpighian tubules; OV: ovary; TE: testis. The data shown are the mean of the relative expression ± standard error (S.E.) (*n* = 3), normalized to *RPS3* transcript levels. Different letters (a, ab, b) above the bar represent a significant difference after ANOVA (least significant difference, LSD, *p < 0.05*).

According to the standard curves generated by the serial diluted cDNA, the primer efficiencies were 96.2% and 97.8% for *TcTβH* and *RPS3*, respectively. As indicated by the qRT-PCR, *TcTβH* was detected across all eight tested developmental stages (Figure 3A), and the highest expression was observed in the old larvae (OL), which was significantly higher than those at any other stages. However, there were not any significant differences among the other stages. For the spatial expression profile of *TcTβH* among the seven different tissues, 5-day-old virgin adults were tested (Figure 3B) and the *TcTβH* transcript was mainly detected in the CNS. Furthermore, a little transcription was detected in the fat body and ovary. No detectable signal (*C*t value was >35) was observed in the other tissues.

### 2.3. OA and TA Measurements and Mobility Assay

**Figure 4:**
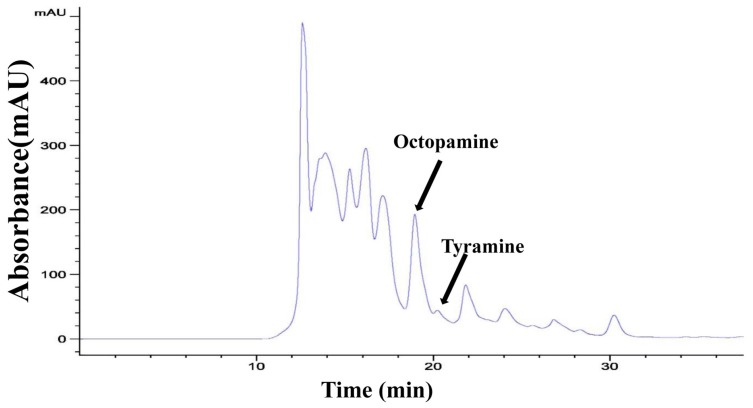
The prominent peak retention time of samples by HPLC with a C30 column. The black arrows are octopamine and tyramine.

We constructed reliable standard curves for OA and TA quantification, as well as the standard curve of Bicinchoninic Acid (BCA) protein for sample calibration. The linear regression equations are provided in Figures S1–S3. According to the results of HPLC, the prominent peak retention times of standard OA and TA were at 18.7 min and at 20.2 min, respectively. The OA and TA in the beetle samples were calculated based on the retention times of standard gradients (Figure 4).

**Figure 5:**
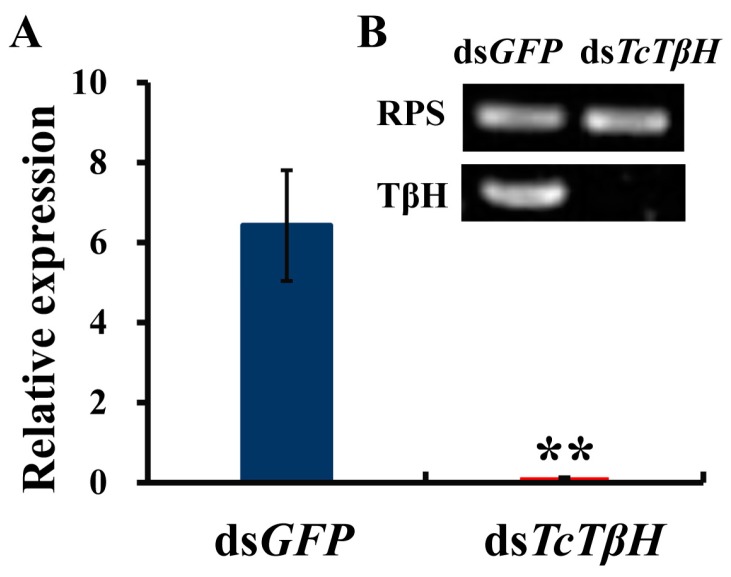
The RNA interference (RNAi) of *TcTβH*. (**A**) The RNAi efficiency was tested by qRT-PCR (the result comes from software of qbase). Data were obtained by analyzing three independent groups of four individuals (two females; two males) per group; (**B**) The confirmation of RNAi efficiency by RT-PCR. Double asterisks represent a significant difference by an independent *t* test (*n* = 3, **, *p* < 0.01), the target gene of *TcTβH* was carried out with 32 cycles and the reference gene *TcrpS* was carried out with 27 cycles.

**Figure 6:**
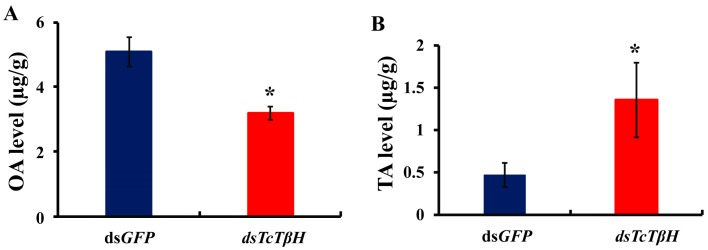
The level of octopamine (OA) and tyramine (TA) by HPLC. (**A**) The detection of the OA level by HPLC after RNAi; (**B**) The detection of the TA level by HPLC after RNAi. The asterisk represents a significant difference by an independent *t* test (*n* = 3, *, *p* < 0.05).

**Figure 7:**
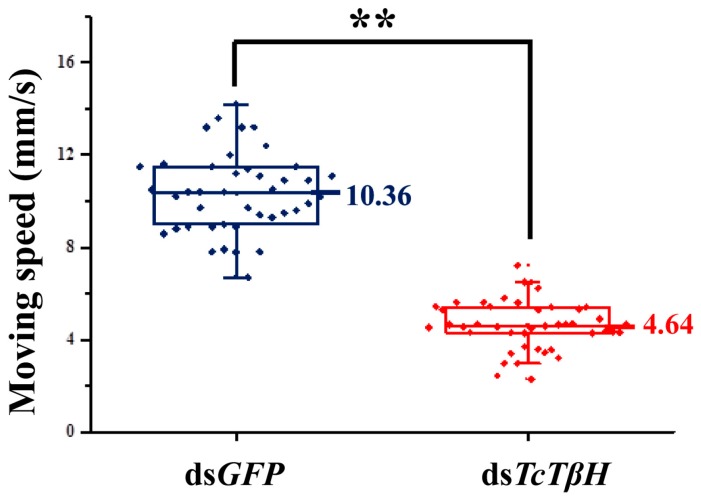
Effect of RNAi of *TcTβH* on the crawling speed (in millimeters per second) of *T. castaneum*. The red box plot shows the moving speed of double-stranded RNA (dsRNA)-treated adults, while the blue box plot shows the controls. Double asterisks represent a significant difference by an independent *t* test (*n* = 100, **, *p* < 0.01).

The silencing efficiency was measured by the qRT-PCR, showing that the pupal injection of ds*TcTβH* significantly suppressed its expression in adults (Figure 5A) and the transcription was reduced by 98%. The significant reduction was further confirmed by regular RT-PCR. The agarose gel electrophoresis showed a very faded band in the ds*TcTβH*-injected group, while the control group showed a very bright band (Figure 5B). Compared with the ds*GFP*-injected beetles, the average OA level in the ds*TcTβH*-injected beetles decreased by 40% (from 5.10 to 3.18 μg/g protein) (Figure 6A). Meanwhile, the average content of TA in beetles was increased approximately two-fold (from 0.49 to 1.41 μg/g protein) (Figure 6B). Furthermore, the average crawling speed of ds*TcTβH*-injected beetles was significantly reduced by 55% to 4.64 mm/s compared to 10.36 mm/s in the control (Figure 7).

## 3. Discussion

With the well-annotated genome of *T. castaneum*, we were able to investigate the important enzyme-TβH in the octopaminergic biosynthesis pathway. In the past, TβH has been identified in various species, such as *Drosophila melanogaster*, *Acyrthosiphon pisum* [19], *Bombyx mori* [20], *Schistocerca gregaria* [21], and *Periplaneta americana* [14]. Multiple sequence alignments with different species revealed that TβH has three main domains, including DOMON-DOH, Cu2-monooxygen and Cu2-monoox-C. This indicates that TβH is highly conserved across the species, implying that it may have a similar physiological function in different insects.

*TcTβH* was predominantly expressed in the larval stage (Figure 3A). A similar expression pattern of TβH was found in the copepod crustacean *Calanus finmarchicus*, which also exhibited a higher expression in larvae [22]. Based on these results, we speculated that TβH plays an important role during the larval stage in *T. castaneum*. In *Drosophila*, it showed high expression in the brain and ventral nerve cord [23]. In addition, *SgTβH* was found to be highly expressed in the CNS and this was consistent with the observation that TβH was localized in the presynaptic terminals of the CNS [21]. Our study showed a similar result, namely a CNS-specific expression of *TcTβH* (Figure 3B), indicating that TβH is functioning in the CNS, where OA is produced.

It has been established that OA and TA are monoamines synthesized from the same precursor—the amino acid tyrosine. OA is the end product in the synthesis pathway, while TA is an intermediate product required for OA synthesis [1]. When knocked down the TβH, the reaction from TA to OA would be disrupted, resulting in a decreased level of OA [24]. However, TA could usually be synthesized from tyrosine, which resulted in an accumulated TA level. A *Drosophila* TβH knockout strain showed much higher amounts of TA because of the termination of the TA hydroxylation during OA synthesis [1]. In the present study, we found that knockdown of *TcTβH* led to reduced OA and accumulated TA in *T. castaneum* (Figure 6). Our data confirmed that TβH plays an essential role in the biosynthesis of OA in *T. castaneum*. In this study, we did not quantify dopamine (DA) and other biogenic amines by HPLC. Although the peak and retention time of standard OA and TA matched very well with samples, we still could not exclude the possibility that there were other biogenic amines in our measurement.

It has been reported that OA modulates locomotion in insects. For example, octopaminergic neurons are thought to modulate muscle metabolism, which is related to flight in insects [25]. OA is involved in modulating the dance behavior of honey bees [26]. According to a study in *D. melanogaster*, OA could modulate the adaptive response to repetitive bouts of hermetic endurance exercise [10] and is required for the fight-fight response in *Drosophilia*. In locusts, OA could promote activation of flight circuitry and recruitment of leg motor units in the flight motor score [7]. We consistently found in this study that beetles with knocking down of *TcTβH* showed significantly decreased mobility. Our data clearly demonstrated that OA modulates locomotion in beetles.

In summary, we demonstrated that TβH serves as a β-hydroxylase which converts TA to OA in *T. castaneum*. It balances the amount of OA and TA by hydroxylation of the TA to produce OA. After knockdown of *TcTβH*, the beetles have a significantly decreased crawling speed. Therefore, we concluded that *TcTβH* controls the biosynthesis amount of OA in the CNS, which in turn is modulating the mobility of the beetles. However, further investigation is needed to understand how the octopaminergic signaling system modulates the beetle’s locomotion. Our results provided basic information on the key genes in the OA biosynthesis pathway in beetles, and expanded knowledge of the physiological functions of OA in insects. In addition, the results will help in further exploration of the complex network of the octopaminergic signaling system regulating locomotion in insects.

## 4. Materials and Methods

### 4.1. Insects

The laboratory colony of *T. castaneum* was fed a diet consisting of wheat flour and brewer’s yeast (10:1) in the Key Laboratory of Entomology and Pest Control Engineering, Southwest University, Chongqing, China. The beetles were reared in standard laboratory conditions of 30 °C, 70 ± 5% relative humidity with a photoperiod regime of 16:8 (L:D) [27]. In this study, all the experimental insects were the Georgia-1 (GA1) strain of *T. castaneum*, originally from Dr. Richard Beeman, and insects were presented by Dr. Yujie Lu in Henan University of Technology, Zhenzhou, China.

### 4.2. Molecular Cloning and Sequences Analysis

Total RNA was extracted from the whole body of four adult beetles with a sex ratio of 1:1 using TRIzol reagent (Invitrogen Life Technologies, Carlsbad, CA, USA), and was treated with RNase-free DNase (Promega, Madison, WI, USA) to remove the genomic DNA based on the manufacturer’s protocol and then followed by a phenol–chloroform extraction. The concentration of RNA was determined by Nanodrop One (Thermo Fisher Scientific, Waltham, MA, USA), while the integrity of RNA was checked by 1.0% agarose gel electrophoresis. First-strand cDNA was synthesized using 1 μg of the total RNA with the GoScript Reverse Transcription System (Promega) for qRT-PCR following the manufacturer’s protocol.

PCR was carried out using the high fidelity DNA polymerase PrimeSTAR (Takara, Dalian, China) to amplify the ORF of *TcTβH*. Primers were designed based on the genome data of *T. castaneum* with Primer Premier 6.0 (Table S1). The PCR procedure was as follows: initial denaturation at 98 °C for 2 min, followed by 35 cycles at 98 °C for 10 s, 56 °C for 5 s and 72 °C for 90 s, and a final extension at 72 °C for 5 min. PCR products were purified and cloned into a pGMT Easy vector (Promega, Madison, WI, USA). Positive clones were sequenced (Invitrogen, Shanghai, China).

### 4.3. Quantitative Real-Time PCR

Different tissue samples and insects at different developmental stages were collected for the expression profiling of *TcTβH*. The beetles at different developmental stages including early eggs (EE), old eggs (OE), early larvae (EL), old larvae (OL), early pupa (EP), old pupa (OP), early adults (EA), and old adults (OA) were prepared as previously described [28]. Different tissues from 7-day-old virgin adults, including from the CNS (containing the brain, thoracic and abdominal ganglia), Malpighian tubules, midgut, hindgut, female ovary and male testis were dissected in ice 1◊phosphate-buffered saline (PBS, pH = 7.4). The extraction of total RNA and first-strand cDNA synthesis was performed as mentioned above.

The gene-specific primers of quantitative real-time PCR (qRT-PCR) were shown in Table S1. The qRT-PCR was performed with an ABI 7500 Real-Time PCR System (Applied Biosystems, Foster City, CA, USA) in a 10 μL reaction system, containing 5 μL of GoTaq qPCR Master Mix (Promega), 3.9 μL of nuclease-free water, 0.5 μL of cDNA samples (approximately 400 ng/μL), and 0.3 μL of each primers (10 μM). The procedure of the reaction included an initial denaturation at 95 °C for 2 min, followed by 40 cycles at 95 °C for 15 s and 60 °C for 30 s. A melting curve analysis from 60 to 95 °C was performed to ensure the specificity. The gene ribosomal protein S3 (rpS3, GenBank accession number: CB335975) was used as an internal reference gene because of its stable expression for the temporal and spatial distribution of *T. castaneum*. All experiments were performed with three biological replications, and the relative expression levels were calculated using the 2^−△△^*^C^*^t^ method [29]. In this study, the old pupa (OP) served as the calibrator for the developmental expression profiling, while the midgut was the calibrator for the tissues-specific expression profiling. The data were analyzed with SPSS16.0 using one-way analysis of variance (ANOVA) followed by least significant difference to separate means.

### 4.4. RNA Interference

Gene-specific primers (Table S1) tailed with T7 promotors were used to amplify the target region for the synthesis of double strand RNA (dsRNA) of *TcTβH*. The amplified products were used as the templates for the synthesis of ds*TcTβH* using the Transcript Aid T7 High Yield Transcription Kit (Thermo Fisher Scientific) based on the manufacturer’s instructions. The integrity of dsRNA was tested by the 1% agarose gel electrophoresis, and the concentration was measured by a Nanodrop One spectrophotometer (Thermo Fisher Scientific). The dsRNA were diluted with PBS (pH = 7.0) to a final concentration of 3000 ng/μL.

Fresh pupae (<24 h old) were employed in the RNAi experiment. Two-hundred nanograms of ds*TcTβH* or ds*GFP* were injected into the junction of the second and third abdominal segment using a Nanoject II microinjector (Drummond Scientific, Broomall, PA, USA). After dsRNA injection, the locomotion assay was conducted using the 5-day-old virgin adults. Meanwhile, four adults (including two males and two females) were pooled together for the RNAi assessment by both qRT-PCR and RT-PCR. The method of qRT-PCR was performed as described above. The RT-PCR was carried out with 32 cycles for the target gene *TcTβH*, with 27 cycles for the reference gene *TcrpS3*. For the RNAi, ds*GFP*-injected beetles served as control.

### 4.5. Mobility Assay

After RNAi, the motility assay was conducted with a Syntech TrackSphere LC-300 (Syntech, Hilversum, The Netherlands) based on the user’s instructions. The Syntech LC-300 is functionally consistent with the locomotion compensator for insects first performed by Kramer [30]. The diameter of the sphere was 30 cm and the crawling tracks of each adult beetle could be recorded by the camera which was attached to the sphere. Before the assay, the parameters of the LC-300 were adjusted using a dark spot on a piece of paper which can be evaluated on the video. When the individual adult was placed in the same place and started walking, the sphere moved toward the opposite direction with the same speed as the beetles. In this assay, each adult beetle was tested for a duration of 2 min, then the average speed of the individuals was calculated. At least 45 adult beetles for the ds*TcTβH*-injected group and also for the control group were tested. The data were analyzed in SPSS 16.0 using the independent sample *t* test.

### 4.6. OA and TA Detection by High Performance Liquid Chromatography (HPLC)

Beetles were rinsed in cold phosphate buffer (0.2 M Na_2_HPO_4_:NaH_2_PO_4_:H_2_O = 36:14:50) and frozen in liquid nitrogen. Subsequently, they were homogenized in a buffer (10% glycerol, 1 mM PMSF, 1 mM/L EDTA dissolved into phosphate buffer, 5 mL per 1 mg beetles) with a mortar-grinder (Coyote Bioscience, 0105003; Beijing, China). The homogenate was centrifuged at 12,000× *g* for 15 min. If the supernatant was still muddy, appropriate Al_2_(SO4)_3_ was added into it. The supernatant was taken and repeatedly centrifuged several times until it could be filtered through a 0.22 μm membrane. The supernatant was kept in the −80 °C freezer upon use.

The chromatographic separation of the supernatant was carried out with a C30 (250 × 4.6 mm, 5 μm) reverse-phase analytical column (YMC, 121FA70201; Shanghai, China). The supernatant was transferred into HPLC vials. Each vial contained a 200 μL gradient, and they were eluted by the mobile phase of 50% A:50% B (A: acetonitrile, B: 3‰ formic acid in water) with a 0.2 mL/min flow rate at 30 °C. The gradients were detected at 274 nm, and each sample was detected with 20 μL for 60 min. The TA and OA were identified by the appearance time of prominent peaks and the content of TA and OA were quantitatively determined by the peak area using Agilent 1260 LC (Agilent Technologies, Santa Clara, CA, USA).

Serial dilutions of OA and TA standards (Sigma, St. Louis, MO, USA) were used to construct the standard curves for their quantification. As for OA, the different concentrations included 160, 80, 60, 40 and 30 μg/mL, while the concentration gradient of TA was 125, 62.5, 31.25, 15.625 and 7.8125 μg/mL. The linear regressions were obtained by plotting the peak area and the content of standard gradients. The homogenate samples were calibrated by the protein level determined using a BCA Protein Assay Kit (Beyotime, Haimen, Jiangsu, China) with a microplate Spectrophotometer (Bio-Rad, Hercules, CA, USA). The standard curve of protein was determined by a serial dilution of the standard protein (0, 0.025, 0.05, 0.1, 0.2, 0.3, 0.4 and 0.5 mg/mL), and the linear regressions were obtained by plotting the optical density and the content of standard protein. The calibration curve equations and the corresponding determination coefficients (*R*^2^) were calculated using all data.

All the experiments were conducted with three biological replications. Twenty beetles were used in each replication for quantification of OA and TA. All the data were analyzed with SPSS 16.0 using an independent sample *t* test.

## Supplementary Materials

Supplementary materials can be found at http://www.mdpi.com/1422-0067/19/3/846/s1.
