# Supplementary material for: How Tyramine β-Hydroxylase Controls the Production of Octopamine, Modulating the Mobility of Beetles
Source: Int J Mol Sci. 2018 Mar 14;19(3):846. doi: 10.3390/ijms19030846 (PMC5877707; doi:10.3390/ijms19030846)
Supplement: Supplementary file 1 [file ijms-19-00846-s001.pdf]

Supplementary

How Tyramine β-Hydroxylase Controls the Production of Octopamine, Modulating the Mobility of Beetles

Li Xu [1,2], Hong-Bo Jiang [1,2], Xiao-Feng Chen [1,2], Ying Xiong [1,2], Xue-Ping Lu [1,2], Yu-Xia Pei [1,2], Guy Smagghe [1,2,3] and Jin-Jun Wang [1,2,*]

1 Key Laboratory of Entomology and Pest Control Engineering, College of Plant Protection, Southwest University, Chongqing 400715, China; Xuli940208@163.com (L.X.); jhb8342@swu.edu.cn (H.-B.J.); 18883375009@163.com (X.-F.C.); xiongying842620@163.com (Y.X.); luxueping91@163.com (X.-P.L.); pyx20830@163.com (Y.-X.P.); guy.smagghe@ugent.be (G.S.)

2 Academy of Agricultural Sciences, Southwest University, Chongqing 400715, China

3 Department of Crop Protection, Ghent University, 9000 Ghent, Belgium

* Correspondence: wangjinjun@swu.edu.cn; Tel.: +86-23-6825-0255; Fax: +86-23-6825-1269

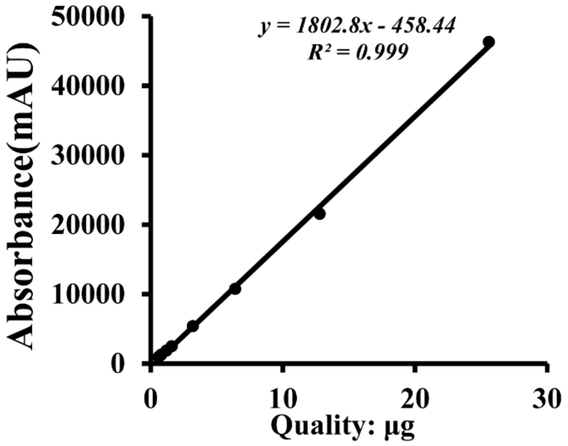

Figure S1. The stand curve of standard OA by HPLC. The linear regression equation of standard OA contain the quality of 25.6, 12.8, 6.4, 3.2 and 1.6 μg.

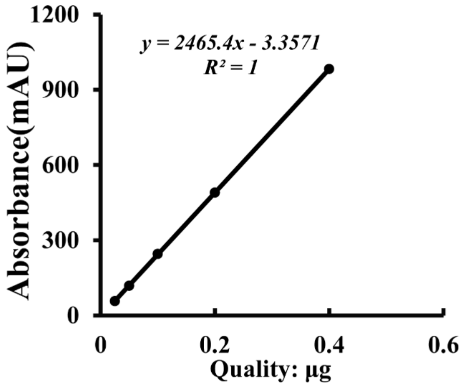

Figure S2. The stand curve of standard TA by HPLC. The linear regression equation of standard TA contain the quality of 20, 10, 5, 2.5 and 1.25 μg.

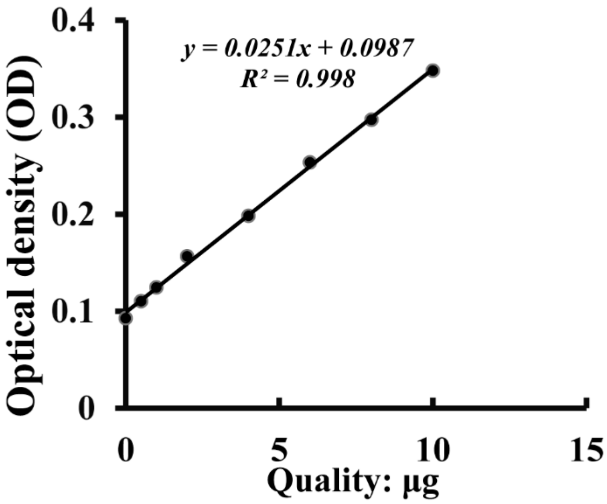

Figure S3. The stand curve of standard protein by microplate reader. The linear regression equation of standard protein contain the quality of 0, 0.5, 1, 2, 4, 6, 8 and 10 mg.

Table S1. Primer sequences of *TcTβH* used for cloning, quantitative real-time PCR and double stranded RNA (dsRNA) synthesis.

**Experiments**	**Primers and sequences（5′-3′）**	**Amiplification Efficiency**	**Product length**
Full-length Confirmation	*TβH-F:* GAAAGATGCGCTTTCTTATC<br>*TβH-R:* TTTTTTAACATGTTCGTGGG	-	1730
qPCR analysis	qPCR-*TcTβH -F:* GGGCACCACCTTTCACATAT<br>qPCR-*TcTβH-R:* CGATAACTCCAGCGTCCATT	96.2%	185
	qPCR-*RPS –F*: CGCATTCATGGTTGATAACG<br>qPCR- *RPS –R*: GGGCACCAAGTTAGTCTGGA	97.8%	186
dsRNA synthesis	ds*TcTβH -F:* taatacgactcactataggCACTGGGATTATACGGCTGGA<br>ds*TcTβH-R:* taatacgactcactataggAACCTTCACTACTACTCGGAATATG	-	493
	ds*GFP -F:* taatacgactcactataggCAGTTCTTGTTGAATTAGATC<br>ds*GFP -R:* taatacgactcactataggTTTGGTTTGTCTCCCATGATG	-	436

TβH, tyramine beta hydroxylase; RPS, ribosomal protein S3; dsRNA, double strand RNA; F, forward; R, reverse; Tc, *Tribolium castaneum*; The lower case letters of dsRNA sequence are T7 promotor sequences for efficient in vitro transcription in dsRNA synthesis.

Table S2. Sequences and relevant information of TβH used for multiple sequence alignment and phylogenetic analysis.

Genes	Accession no.	Species
AmTβH	NP_001071292.1	*Apis mellifera*
HlTβH	XP_017788112	*Habropoda laboriosa*
EmTβH	XP_017755374.1	*Eufriesea mexicana*
MyTβH	KOX68655	*Mizuhopecten yessoensis*
CfTβH	XP_011268213.1	*Camponotus floridanus*
LnTβH	KMQ92961	*Lasius niger*
FaTβH	XP_011307313	*Fopius arisanus*
TcTβH	XP_974169	*Tribolium castaneum*
NvTβH	AHN85840.1	*Nicrophorus vespilloides*
PaTβH	AFO63080.1	*Periplaneta americana*
GbTβH	BAO52001	*Gryllus bimaculatus*
AaTβH	XP_021707312	*Aedes aegypti*
DmTβH	AHN59467.1	*Drosophila melanogaster*
PrTβH	BAM66418	*Phormia regina*
CcTβH	XP_018400397	*Cyphomyrmex costatus*
PxTβH	KPI95666	*Papilio xuthus*
BmTβH	NP_001243923	*Bombyx mori*
ObTβH	KOB79082.1	*Operophtera brumata*
